# Metformin in women with type 2 diabetes in pregnancy (MiTy): a multi-center randomized controlled trial

**DOI:** 10.1186/s12884-016-0954-4

**Published:** 2016-07-19

**Authors:** Denice S. Feig, Kellie Murphy, Elizabeth Asztalos, George Tomlinson, Johanna Sanchez, Bernard Zinman, Arne Ohlsson, Edmond A. Ryan, I. George Fantus, Anthony B. Armson, Lorraine L. Lipscombe, Jon F.R. Barrett, on behalf of the MiTy Collaborative Group, George Carson, Suzanne Williams, Sheila Kelly, Heather Clark, Diane Donat, Shital Gandhi, Barbara Cleave, Michele Strom, Lois Donovan, Carolyn Oldford, Catherine Young, Brenda Galway, Minnie Parsons, Ariane Godbout, Michele Mahone, Florence Weber, Marie-Josee Bedard, Bi Lan Wo, Sylvie Daigle, Amir Hanna, Maria Wolfs, Leanne De Souza, Robyn Houlden, Adriana Breen, Tina Kader, Luca Cefis, Erin Keely, Josee Champagne, Jennifer Klinke, Julie Lee, Peter Subrt, Francina Carr, Sharon Young, Peter Subrt, Francina Carr, Sharon Young, Sarah Kwong, Heather Rylance, Helene Long, Julie Lambert, Julia Lowe, Anna Rogowsky, Susan Jackson, Sora Ludwig, Laurie Slater, Lori Berard, David McIntyre, Anne Tremellen, Ruth McManus, Sara Meltzer, Natasha Garfield, Shari Segal, David Miller, Karen Coles, Carol Fergusson, Jill Newstead-Angel, Judy Brandt, Thomas Ransom, Darlene Baxendale, Evelyne Rey, Diane Francoeur, Sophie Perreault, Heather Rylance, Dory Sample, David Thompson, Sharon Thompson, John S Weisnagel, Camile Lambert, Valerie-Eve Julien, Marie-Christine Dube, Louise Rheaume, Afshan Zahedi, Grace Lee, Krystyna Szwiega, Asma Qureshi, Siobhan Tobin

**Affiliations:** Mount Sinai Hospital, 60 Murray St., Lebovic Building, Suite 5027, M5T 3L9 Toronto, Ontario Canada; Lunenfeld-Tanenbaum Research Institute, Toronto, Ontario Canada; Department of Medicine, University of Toronto, Toronto, Canada; Sunnybrook Health Sciences Centre Toronto, Toronto, Canada; University Health Network, Toronto, Canada; Institute for Health Policy, Management and Evaluation, University of Toronto, Toronto, Ontario Canada; Sunnybrook Research Institute Toronto, Toronto, Ontario Canada; The Centre for Mother, Infant and Child Research (CMICR), Toronto, Ontario Canada; Department of Paediatics, University of Toronto, Toronto, Canada; University of Alberta, Edmonton, Canada; Dalhousie University, Halifax, Nova Scotia Canada; Women’s College Hospital, Toronto, Ontario Canada

**Keywords:** Diabetes Mellitus, Pregnancy, Pregnancy in diabetics, Metformin, Diabetes Mellitus Type 2, Pregnancy outcome, Randomized controlled trial

## Abstract

**Background:**

The incidence of type 2 diabetes in pregnancy is rising and rates of serious adverse maternal and fetal outcomes remain high. Metformin is a biguanide that is used as first-line treatment for non-pregnant patients with type 2 diabetes. We hypothesize that metformin use in pregnancy, as an adjunct to insulin, will decrease adverse outcomes by reducing maternal hyperglycemia, maternal insulin doses, maternal weight gain and gestational hypertension/pre-eclampsia. In addition, since metformin crosses the placenta, metformin treatment of the fetus may have a direct beneficial effect on neonatal outcomes. Our aim is to compare the effectiveness of the addition of metformin to insulin, to standard care (insulin plus placebo) in women with type 2 diabetes in pregnancy.

**Methods:**

The MiTy trial is a multi-centre randomized trial currently enrolling pregnant women with type 2 diabetes, who are on insulin, between the ages of 18–45, with a gestational age of 6 weeks 0 days to 22 weeks 6 days. In this randomized, double-masked, parallel placebo-controlled trial, after giving informed consent, women are randomized to receive either metformin 1,000 mg twice daily or placebo twice daily. A web-based block randomization system is used to assign women to metformin or placebo in a 1:1 ratio, stratified for site and body mass index. The primary outcome is a composite neonatal outcome of pregnancy loss, preterm birth, birth injury, moderate/severe respiratory distress, neonatal hypoglycemia, or neonatal intensive care unit admission longer than 24 h. Secondary outcomes are large for gestational age, cord blood gas pH < 7.0, congenital anomalies, hyperbilirubinemia, sepsis, hyperinsulinemia, shoulder dystocia, fetal fat mass, as well as maternal outcomes: maternal weight gain, maternal insulin doses, maternal glycemic control, maternal hypoglycemia, gestational hypertension, preeclampsia, cesarean section, number of hospitalizations during pregnancy, and duration of hospital stays. The trial aims to enroll 500 participants.

**Discussion:**

The results of this trial will inform endocrinologists, obstetricians, family doctors, and other healthcare professionals caring for women with type 2 diabetes in pregnancy, as to the benefits of adding metformin to insulin in this high risk population.

**Trial registration:**

ClinicalTrials.gov Identifier: no. NCT01353391. Registered February 6, 2009.

**Electronic supplementary material:**

The online version of this article (doi:10.1186/s12884-016-0954-4) contains supplementary material, which is available to authorized users.

## Background

### Burden of type 2 diabetes in pregnancy

The incidence of type 2 diabetes is rising worldwide at a remarkable rate [1]. This rise has been accompanied by a decrease in the age of onset of diabetes, especially in women, [2] with a resultant escalation in the rate of type 2 diabetes in pregnancy [3, 4]. In our study of deliveries in Ontario, the prevalence of preexisting diabetes (type 1 and type 2 diabetes) in pregnancy doubled in the last 14 years, from 7 per 1,000 deliveries in 1996 to 15 per 1,000 deliveries in 2010 [4]. For women over the age of 30 years, almost 2 % of women who deliver have pre-existing diabetes in pregnancy. Studies have shown that this increase is largely driven by a rise in type 2 diabetes in pregnancy [5, 6].

The offspring of women with type 2 diabetes continue to have increased rates of perinatal morbidity and mortality [4]. Infants of mothers with type 2 diabetes have high rates of being born large for gestational age (LGA) (28–56 %) and macrosomic (>4 or 4.5 kg) (8–9.3 %) [7–12]. Macrosomia is associated with increased rates of perinatal asphyxia, meconium aspiration, hypoglycemia, shoulder dystocia, brachial plexus injury, skeletal injuries, and fetal death [13–17]. Poor glycemic control in mothers with diabetes leads to an increased risk of severe respiratory distress syndrome, low Apgar scores, neonatal hypoglycemia and neonatal intensive care unit (NICU) admissions [7–12].

Women with type 2 diabetes have high rates of maternal morbidity including gestational hypertension, preeclampsia (17–19 %) and caesarean delivery (36–53 %) [7–12]. They are often obese and have marked insulin resistance which worsens during pregnancy, leading frequently to very high insulin requirements. Taking such large doses of insulin is uncomfortable, expensive and challenging. Absorption kinetics may be altered when very large doses are delivered to one site, leading to a failure to reduce postprandial hyperglycemia, but with later hypoglycaemia once the insulin is absorbed. When receiving large doses, patients complain of pain at the site of injection leading to compliance issues and poor glycemic control. Very high insulin doses can also cause excessive weight gain in already obese women. In a recent study of obese women with type 2 diabetes, those with maternal weight gain < =5 kg had lower rates of large for gestational age and less perinatal morbidity than those who gained more weight [18].

### Anticipated benefits of adding metformin

Metformin is a biguanide that has been used for many years in the management of type 2 diabetes. Metformin has been shown to lower glucose levels by inhibiting mitochondrial glycerophosphate dehydrogenase, thus reducing glucose production by the liver [19]. Metformin improves insulin resistance by increasing glucose transport in skeletal muscle and adipocytes, and increases endogenous glucagon-like peptide 1 concentration [20].

Based on the pharmacological benefits in individuals with diabetes, there are sound theoretical reasons to use metformin in pregnant women with type 2 diabetes.

1. By reducing hepatic gluconeogenesis and improving insulin sensitivity, metformin may improve maternal glycemic control leading to less hyperinsulinemia, LGA and their associated complications.
2. Metformin use with insulin may lead to a reduction of maternal insulin doses required for good glycemic control leading to better compliance and reduced maternal weight gain. The additional use of metformin in pregnant women with type 2 diabetes has been shown to decrease insulin requirements by up to 60 % [21]. In the Metformin in Gestational Diabetes trial, a randomized trial of metformin versus insulin in women with gestational diabetes mellitus (GDM), women on metformin required significantly less insulin than women taking insulin alone and gained significantly less weight from enrolment to 37 weeks gestation compared to women on insulin (0.4 kg vs. 2.0 kg, *p* < 0.001) [22]. In a recent meta-analysis of randomized controlled trials of metformin use in women with GDM, there was significantly less maternal weight gain in women taking metformin as compared with insulin [23]. It has been postulated that a reduction in weight gain with metformin treatment may be due to decreased appetite, and a reduction in hyperinsulinemia associated with reduced insulin resistance [22].
3. Metformin may reduce insulin resistance in the infant (as metformin crosses the placenta). Although there is as yet no information on the action of metformin in the fetus, we hypothesize that metformin may improve fetal insulin sensitivity. There is evidence that LGA infants of women with diabetes are born with insulin resistance [24] and we hypothesize that a reduction in insulin resistance may lead to decreased rates of hyperinsulinemia, macrosomia, birth injury, neonatal hypoglycemia, and NICU admissions. In a meta-analysis of randomized trials comparing metformin to insulin therapy in women with GDM, infants of mothers with GDM taking metformin had a lower birth weight compared to controls (*p* = 0.05) [25]. Lower insulin levels in the fetus may also result in less neonatal hypoglycemia and fewer NICU admissions. In this meta-analysis, women taking metformin showed reduced neonatal hypoglycemia, although preterm birth was slightly increased [25]. In a recent meta-analysis of women with polycystic ovary syndrome, metformin use throughout pregnancy decreased the risk of early pregnancy loss, GDM, preeclampsia and preterm birth [26].

In addition, exposure to intrauterine hyperglycemia has been associated with an increased risk of diabetes and obesity in adolescence and adulthood [27, 28]. Intrauterine exposure to metformin may reduce this ‘intrauterine programming’ and lower the long-term risk of diabetes and obesity in these children. A recent study of 2 year old infants exposed to metformin during the Metformin in Gestational Diabetes trial, investigators found that infants exposed to metformin in utero had increased subscapular and biceps skinfolds compared to unexposed infants, although total body fat was similar [29]. The authors speculate that this may represent a healthier distribution of fat, with increased peripheral fat deposition and less visceral fat deposition. This finding requires confirmation.

Evidence from other studies:

Only one small randomized controlled trial has looked at the addition of metformin to insulin in women with type 2 diabetes in pregnancy. In an open label study of 90 women with insulin resistance and GDM or type 2 diabetes (breakdown of numbers with GDM or type 2 diabetes not specified), a reduction in neonatal hypoglycemia and NICU admissions were found when metformin was added to insulin [30]. In addition to the small sample size, this study had several limitations, including late randomization (as late as 34 weeks gestation), failure to employ an intention-to-treat analysis, and lack of blood glucose self monitoring.

The aim of the present study is to compare the effectiveness of the addition of metformin to insulin to standard care (insulin alone) in women with type 2 diabetes in pregnancy.

## Methods/Design

### Overall study design

This is a multicentre, randomized, double-masked, placebo-controlled trial in women with type 2 diabetes in pregnancy. Participating sites comprise 21 centres in Canada and 1 centre in Australia. Women with type 2 diabetes in pregnancy, between 6^+0^ and 22^+6^ weeks gestation who are currently on insulin, are eligible. Eligible women are randomized to receive either metformin or placebo, to be added to their usual insulin regimen. Patients, caregivers and outcome assessors are blinded to which intervention is received. Baseline data are obtained at the time of randomization and at 4 week intervals until delivery. The primary outcome is a composite of perinatal outcomes. The analysis will be conducted using an intention-to-treat approach.

Primary Research Question: Among pregnant women with diagnosed type 2 diabetes mellitus, does the addition of metformin to a standard regimen of insulin, increase or decrease the incidence of adverse perinatal outcomes as defined by a composite of pregnancy loss, preterm birth, birth injury, respiratory distress, neonatal hypoglycemia and NICU admission longer than 24 h, compared with women treated with insulin plus placebo?

This trial is funded by the Canadian Institute of Health Research MOP 106678. The metformin and placebo tablets have been donated by Apotex Inc.

### Participants

#### Eligibility criteria

Pregnant women with a live, singleton fetus, with type 2 diabetes between the ages of 18–45, currently on insulin, with a gestational age of 6^+0^–22^+6^ weeks are eligible to participate. Women are eligible if they have undiagnosed type 2 diabetes prior to 20 weeks gestation (as defined by 2 of any of the following: fasting glucose levels ≥ 7.0 mmol/L (126 mg/dl) or glycated hemoglobin values of ≥0.065 (48 mmol/mol) performed in a laboratory using a method that is standardized to the Diabetes Control and Complications Trial assay, or a two hour ≥ 11.1 mmol/L (200 mg/dl) on a 75 g Oral Glucose Tolerance Test).

All women require a dating ultrasound to confirm gestational age, viability and rule out multiples. Gestational age is based on the last menstrual period, provided there is a ≤5 day discrepancy with ultrasound dates in the first trimester and ≤10 day discrepancy with ultrasound dates in the second trimester. If the dates from the last menstrual period are outside these limits, the ultrasound dates are used as the best estimate of gestational age. Women who are on oral hypoglycemic agents (including metformin) are taken off and started on insulin prior to randomization.

#### Exclusion criteria

Women are excluded if they were diagnosed with type 2 diabetes after 20 weeks gestation, have Type 1 diabetes, have a known intolerance to metformin, have current significant gastrointestinal problems such as severe vomiting requiring intravenous fluids or hospitalization, active Crohn’s or colitis, presence of acute or chronic metabolic acidosis, including diabetic ketoacidosis, a history of diabetic ketoacidosis or history of lactic acidosis. Women with excessive alcohol intake, acute or chronic, those with congestive heart failure or a history of congestive heart failure and those with contraindications to metformin use are excluded. These include renal insufficiency (defined as serum creatinine of greater than 130 umol/L or creatinine clearance <60 ml/min, moderate to severe liver dysfunction (defined as liver enzymes (aspartate aminotransferase and alanine aminotransferase) greater than 3 times the upper limit of normal), shock or sepsis, and previous hypersensitivity to metformin. Women who have a fetus with a known potentially lethal anomaly are excluded. Information regarding congenital anomalies diagnosed after randomization is recorded. Women with known higher order pregnancies (twins, triplets, etc.) and those with prior participation in this trial are excluded.

### Recruitment

Women who meet eligibility criteria and who are being seen in obstetric or endocrine clinics are approached about participation in the trial and given information pamphlets describing the study. Those women who are interested are asked to sign a consent form. Women are told that participation is voluntary and that they can withdraw at any time without affecting their clinical care. At baseline a medical history is obtained along with other baseline demographics and concomitant medications including insulin regimen and dose (see Table 1). Weight, height and blood pressure are measured and recorded and blood samples are assessed locally for glycemic control, and renal parameters (serum creatinine and albumin to creatinine ratio).

**Table 1:** Data Collection Schedule

	*At Entry*	*At Each Prenatal Visit*	*Every 4 Weeks*	*At End of Pregnancy*
Demographics:				
Age	x			
Parity	x			
Duration of Diabetes	x			
Diabetes Complications	x			x
Hypertension	x	x		x
Ethnicity	x			
HbA1c	x		x	x
Serum Creatinine	x			At 36 weeks GA
Hypoglycemic Episodes	x	x		x
Maternal weight, height	x	x		x
Maternal insulin dose	x	x		x
Insulin type used	x			
No. of Prenatal Visits				x
Delivery Information				x
Preeclampsia				x
Cord blood for c-peptide and cord gases				x
Infant Data				x
Neonatal skin folds				x

### Randomization

**Fig. 1:**
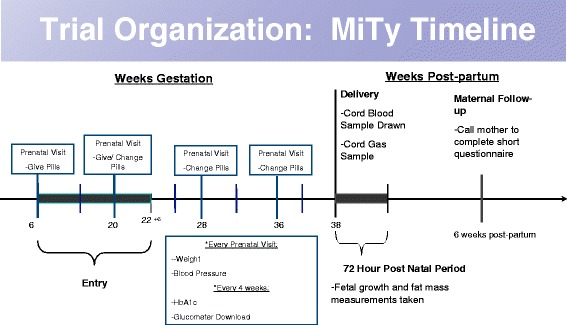
MiTy timeline of visits, measurements, blood samples and follow-up

Randomization occurs between 6^+0^ and 22^+6^ weeks gestation (see Fig. 1). Participants are assigned via the Centre for Maternal, Infant and Child Research trial website at Women’s College Hospital, to receive metformin or placebo in a 1:1 ratio, in randomly chosen block sizes. The trial randomization stratifies for site and body mass index (<30 or ≥30 kg/m^2^) calculated at the time of randomization.

### Intervention

Metformin is provided in 500 mg tablets. The placebo tablets have identical appearance, taste, labelling and expiry dates, and are dispensed and administered in the same manner. Metformin or placebo is self-administered by the participants in addition to their usual insulin regimen. The Site Investigator reduces insulin doses if needed. The treatment period continues from the morning after the randomization visit until delivery.

Compliance is enhanced by the fact that metformin is an oral agent; however, women given metformin may have an increased incidence of gastrointestinal side-effects such as abdominal cramps and diarrhea. This is minimized by introducing the medication gradually, and decreasing the dose to the best tolerated dose for those who cannot tolerate the full dose. A potential for unmasking may occur if the insulin dose drops or the participant experiences diarrhea. Both of these effects can be minimized by the gradual increase in dosage of the study medication. Compliance is defined as taking 80 % of the prescribed pills. Any co-intervention should be balanced as all caregivers are masked to treatment group.

Prior to confirmation of pregnancy some of the women may have been on metformin or other glucose lowering agents for the treatment of their type 2 diabetes, and this medication may have been continued up to the time of randomization. Information regarding the use of metformin and other glucose lowering agents prior to randomization is collected.

### Follow-up visits and data collection

Prenatal Visits occur every 4 weeks after randomization. At each visit changes in medical history and medications are reviewed (including insulin type and dose), study medication compliance is assessed. Adverse events, episodes of severe hypoglycaemia, and hospitalizations are documented. Weight and blood pressure are obtained and glucometer data are downloaded. Local HbA1c (glycated haemoglobin) and albumin to creatinine ratio are obtained and study medication is dispensed (at 14, 22 and 30 weeks gestation). At this time a pill count is done to document compliance.

### Delivery

At delivery cord blood is taken for blood gases (to be done locally) and c-peptide analysis. Cord blood for c-peptide is spun and stored at-80° Celsius, and shipped on dry ice to Mount Sinai Hospital for analysis. Delivery and neonatal outcomes are collected. Neonatal anthropometric measurements including length, weight, head circumference and skinfolds are performed within 72 h of delivery. At 6 weeks postpartum women are asked about neonatal wellbeing and about their satisfaction in the trial. They are asked if they wish to enrol in the MiTy Kids follow up trial where infants of women In the MiTy trial are followed for up to 2 years of age.

### Primary outcome

The primary outcome is a composite defined as the occurrence of one or more of the following: pregnancy loss, preterm birth, birth injury, moderate/severe respiratory distress, neonatal hypoglycemia, and NICU admission > 24 h.

### Secondary outcomes

The secondary outcomes include the individual components of the composite as well as the incidence of large for gestational age infants defined as greater than the 90th percentile for weight, based on the National Canadian fetal growth standards for singleton boys and girls [31]; congenital anomalies; cord blood gas pH <7.0; hyperinsulinemia as measured by elevated cord blood c-peptide > 1.7 ug/L; sepsis; hyperbilirubinemia; shoulder dystocia; fetal fat mass as measured by neonatal anthropometric analysis [32]; maternal weight gain; maternal insulin doses; maternal glycemic control as measured by HbA1c and capillary glucose measurements; maternal hypoglycemia defined as mild (<3.6 mmol/L (65 mg/dL)), symptomatic and asymptomatic or requiring treatment), or severe (loss of consciousness or confusion requiring assistance); incidence of pre-eclampsia, and/or gestational hypertension; number of hospitalizations prior to admission for delivery; the duration of hospital stays for the mother prior to admission for delivery and associated with delivery; rate of caesarean delivery; duration of hospital stay for the infant.

### Avoiding bias

The trial design includes double-masking to avoid bias in the assessment of outcomes. This minimizes co-interventions. Every effort is made to avoid unmasking of participants. In the event that unmasking cannot be avoided, sites notify the MiTy coordinating centre, and the designated programmer securely provides the Site Investigator with the participant’s allocation.

### Sample size and power calculation

The sample size was calculated using the incidence of the composite outcome from the validated Nova Scotia Atlee Perinatal Database^.^ In this database, the incidence of the composite outcome was 62 %. For the purpose of calculating the sample size, a conservative value of 50 % was assumed. A relative risk reduction of 25 % (i.e., from 50 to 37.5 %) was considered clinically relevant. A total of 246 women per treatment group gives 80 % power if the addition of metformin decreases the risk of the composite outcome by 25 % (2-tailed α of 0.05). Accounting for 2 % loss to follow-up, a sample size of 250 per treatment group or 500 in total is planned. If the incidence is as high as 62 %, we have over 90 % power for a relative risk reduction of 25 and 78 % power for a relative risk reduction of 20 %. Although our hypothesis is that the addition of metformin will decrease the event rate of the primary composite outcome, we cannot be sure that we are not causing harm, and therefore we are using a two-sided alpha for the sample size calculation.

### Data analysis

#### Interim analysis and safety considerations

There is one planned interim analysis of the composite outcome after 50 % of subjects (*n* = 246) have completed the Postpartum Visit (telephone interview). Using the O’Brien–Fleming procedure [33], the interim analysis will use a significance level of 0.003 for stopping the trial early. The MiTy coordinating centre will prepare the data for the interim analysis and the analysis will be sent to the statistician without unmasking the groups. After analysis the data will be sent to the Data Safety Monitoring Board (DSMB). The DSMB is made up of experts from endocrinology, obstetrics, epidemiology, and clinical trials methodology. The DSMB will convene by teleconference for the interim analysis. If the overall rate of adverse outcomes is larger than expected, or if there are unanticipated adverse events, the DSMB will have the right to analyze unmasked data.

The Steering Committee receives a summary of all reported serious adverse events (SAEs) at regular scheduled meetings (quarterly). The Steering Committee may be requested to review SAEs (masked) off-cycle as determined by the Working Group. The Working Group is made up of the principal investigator, some members of the Steering Committee, and members of the coordinating centre. If reported (masked) SAEs are of concern to members of the Steering Committee, or if there is an emerging pattern or unexpected frequency of events, the DSMB will be notified and information forwarded to them for review. Otherwise, all adverse events are reviewed by the DSMB at the time of the planned interim analysis.

#### Final Analysis

An “intention-to-treat” analysis will be performed, with subjects analyzed in the group to which they were randomized. The primary analysis will calculate the relative risk (and 95 % CI) of the composite neonatal outcome between the treated and control groups, stratifying by site and BMI group using a log-binomial regression model [34], and test the null hypothesis that the relative risk is equal to 1. We will estimate an overall absolute risk reduction and its 95 % CI and use this to compute a number needed to treat to benefit with metformin to avoid one adverse composite outcome. A similar analysis will be carried out for each of the dichotomous secondary outcomes. Continuous secondary outcomes measured at delivery will be compared using a two sample t-test or, if necessary, a bootstrap hypothesis test. The repeated measurements on HbA1c will be compared between groups using a linear mixed effects model. If important covariates remain imbalanced between treatment groups despite the stratified randomization, these covariates will be added to the log-binomial model and the difference between adjusted and unadjusted relative risks will be examined to assess the impact of this imbalance. All analyses will use a 2-tailed α of 0.05.

### Data management

All data and statistical issues will be managed by the Centre for Maternal, Infant and Child Research and the trial statistician.

### Ethics committee approval

The study was approved by the Mount Sinai Hospital Ethics Review Board (Protocol No. NSP 104011) and by all the participating sites (see Additional file 1) at their local ethics boards.

### Trial steering committee

The MiTy Steering Committee is responsible for the conduct of the trial and meets by teleconference on a quarterly basis.

## Discussion

### Implications of findings

The results of this study will directly inform clinical practice and standards of care, ultimately optimizing short and long-term health outcomes of pregnant women with type 2 diabetes and their children. If the results show improved maternal and fetal/neonatal outcomes using metformin, then physicians treating women with type 2 diabetes in pregnancy will choose to use metformin with insulin as a new standard of care, to reduce serious perinatal morbidity. We may determine whether the addition of metformin is particularly useful in certain subsets of the population i.e., obese women, those on high doses of insulin, and allow us to tailor our treatment to those who would benefit most. Follow-up in MiTy Kids will help determine the long-term outcomes of metformin exposure in-utero and favourable outcomes in offspring will amplify the impetus to use metformin in type 2 diabetes during pregnancy. Adverse outcomes related to metformin without any metabolic or pregnancy outcome benefit will also be important to document and will similarly inform clinical practice. In either case the data from MiTy and MiTy Kids will help clarify clinical practice guidelines.

## Conclusion

Preliminary data from randomized trials comparing metformin to insulin in women with gestational diabetes and one small randomized trial in women with type 2 diabetes suggest metformin may have beneficial effects when added to insulin during pregnancy. However, definitive data on the efficacy of metformin in reducing adverse maternal and fetal outcomes in this population are lacking. No studies have established whether the use of metformin with insulin is beneficial and the growing incidence of type 2 diabetes in pregnant mothers makes this an increasingly relevant and important question. MiTy will provide critical insight into an important therapeutic question which will have an impact on standard of care and clinical practice, with the potential to benefit the health of pregnant mothers with diabetes and their children, immediately and in the long-term.

## Abbreviations

BMI: Body mass index; DSMB: Data Safety Monitoring Board; GDM: Gestational diabetes; HbA1c: Glycated haemoglobin; LGA: Large for gestational age; MiTy: Metformin in women with type 2 diabetes in pregnancy trial; NICU: Neonatal intensive care unit admissions; SAEs: Serious adverse events.

## Additional file

Additional file 1: Collaborating Centres. (DOCX 117 kb)
