# Supplementary material for: Metformin in women with type 2 diabetes in pregnancy (MiTy): a multi-center randomized controlled trial
Source: BMC Pregnancy Childbirth. 2016 Jul 19;16:173. doi: 10.1186/s12884-016-0954-4 (PMC4952061; doi:10.1186/s12884-016-0954-4)
Supplement: Additional file 1: — Collaborating Centres. (DOCX 117 kb) [file 12884_2016_954_MOESM1_ESM.docx]

**Collaborating Centres**

| *Collaborators* | *Collaborating Centres* |
| --- | --- |
| Armson, Anthony and Ransom, Thomas | Izaak Walton Killam Health Centre (IWK),  *Halifax, NS, Canada* |
| Miller, David | Vancouver Island Health Authority,  *Victoria, BC, Canada* |
| Carson, George | Regina General Hospital, Regina Qu'Appelle Health Region,  *Regina, SK, Canada* |
| Clark, Heather | The Ottawa Hospital Civic Campus,  *Ottawa, ON, Canada* |
| Donat, Diane and Feig, Denice | Mount Sinai Hospital,  *Toronto, ON,Canada* |
| Donovan, Lois | Alberta Health Services, University of Calgary,  *Calgary, AB, Canada* |
| Galway, Brenda | Memorial University of Newfoundland, Health Sciences Centre,  S*t.John’s, NL, Canada* |
| Godbout, Ariane | Centre Hospitalier de Université de Montréal, Hôpital St. Luc,  *Montréal, QC, Canada* |
| Hanna, Amir and Wolfs, Maria | St. Michael's Hospital,  *Toronto, ON, Canada* |
| Houlden, Robyn | Kingston General Hospital, Queen’s University,  *Kingston, ON, Canada* |
| Kader, Tina | Jewish General Hospital,  *Montreal, QC, Canada* |
| Keely, Erin | The Ottawa Hospital General Campus,  *Ottawa, ON, Canada* |
| Klinke, Jennifer and Lee, Julie | Royal Columbian Hospital,  *New Westminster, BC, Canada* |
| Kwong, Sarah | Alberta Health Services, Grey Nuns Hospital,  *Edmonton, AB, Canada* |
| Long, Helene | Centre Régional du diabète de Laval, Centre de Santé et de Services Sociaux de Laval,  *Montreal, QC. Canada* |
| Lowe, Julia | Sunnybrook Health Sciences Centre,  *Toronto, ON, Canada* |
| Ludwig, Sora | St. Boniface General Hospital,  *Winnipeg, MB, Canada* |
| McManus, Ruth | St. Joseph's Health Centre,  *London, ON, Canada* |
| Meltzer, Sara and Garfield, Natasha | McGill University Health Centre,  *Montreal, QC,Canada* |
| McIntyre, H David | Mater Clinical School & Mater Medical Research Institute,  Brisbane, Australia |
| Newstead-Angel, Jill | Royal University Hospital,  *Saskatoon, SK, Canada* |
| Rey, Evelyne and Francoeur, Diane | Centre Hospitalier Universitaire (CHU) Sainte-Justine,  *Montreal, QC, Canada* |
| Ryan, Edmond | Alberta Health Services, University of Alberta,  *Edmonton, AB, Canada* |
| Thompson, David | Children and Women’s Health Centre of British Columbia, University of British Colombia,*Vancouver, BC, Canada* |
| Weisenagel, S. John | Centre Hospitalier Universitaire de Québec (CHUQ),  *Quebec City, QC, Canada* |
| Zahedi, Afshan | The Scarborough Hospital,  *Toronto, ON, Canada* |

Ethics Committees That Granted Approval

| Centre | Hospital | REB Name |
| --- | --- | --- |
| 001-001 | Royal University Hospital | University of Saskatchewan Biomedical Research Ethics Board |
| 001-004 | Mount Sinai Hospital | Mount Sinai Hospital Research Ethics Board |
| 001-005 | Children's & Women's Health Centre of BC | UBC Children's & Women's Research Ethics Board |
| 001-006 | Regina General Hospital | Regina Qu’Appelle Health Region Research Ethics Board |
| 001-007 | St. Boniface General Hospital | University of Manitoba Bannatyne Campus Research Ethics Boards |
| 001-008 | IWK Health Centre | IWK Research Ethics Board |
| 001-009 | Sunnybrook Health Sciences Centre | Research Ethics Board of Sunnybrook Health Sciences Centre |
| 001-011 | Kingston General Hospital | Queen’s University Health Sciences & Affiliated Teaching Hospitals Research Ethics Board |
| 001-013 | CHU Sainte-Justine | CHU Sainte-Justine Research Ethics Board |
| 001-014 | The Ottawa Hospital General Campus | Ottawa Hospital Research Ethics Board |
| 001-015 | The Ottawa Hospital Civic Campus | Ottawa Hospital Research Ethics Board |
| 001-017 | Grey Nuns Hospital | University of Alberta- Health Research Ethics Board Biomedical Panel |
| 001-023 | Royal Columbian Hospital | Fraser Health Research Ethics Board |
| 001-026 | McMaster University | Hamilton Health Sciences/McMaster Health Sciences Research Ethics Board |
| 001-030 | St. Michael’s Hospital | St. Michael’s Hospital Research Ethics Board |
| 001-032 | St Josephs Health Care | University of Western Ontario Health Sciences Research Ethics Board |
| 001-033 | Jewish General Hospital | Research Ethics Committee of the Jewish General Hospital |
| 001-035 | McGill University Health Centre | Biomedical A Research Ethics Board of McGill University Health Centre |
| 001-036 | Alberta Health Services | University of Calgary Conjoint Health Research Ethics Board |
| 001-038 | Vancouver Island Health Research Centre | Vancouver Island Health Authority Clinical Research Ethics Board |
| 001-042 | The Scarborough Hospital | The Scarborough Hospital Research Ethics Board |
| 001-043 | Memorial University Health Sciences Center | Memorial University Human Investigation Committee |
| 001-044 | Cite de la Sante de Laval | Cite de la Sante de Laval Comité scientifique et d'éthique de la recherche |
| 001-045 | CHUQ-CHUL | Comité d'éthique de la recherché du CHU de Quebec |
| 001-046 | University of Alberta- Royal Alexander Hospital | University of Alberta, Health Research Ethics Board - Biomedical Panel |
| 001-047 | Hopital St-Luc du CHUM | Comité d’éthique de la recherche du CHUM |
| 004-020 | Mater Mothers Hospital | Mater Health Services Human Research Ethics Committee |
